# ANN Surface Roughness Optimization of AZ61 Magnesium Alloy Finish Turning: Minimum Machining Times at Prime Machining Costs

**DOI:** 10.3390/ma11050808

**Published:** 2018-05-16

**Authors:** Adel Taha Abbas, Danil Yurievich Pimenov, Ivan Nikolaevich Erdakov, Mohamed~Adel Taha, Mahmoud Sayed Soliman, Magdy Mostafa El Rayes

**Affiliations:** 1 Department of Mechanical Engineering, College of Engineering, King Saud University, P.O. Box 800, Riyadh 11421, Saudi Arabia; solimanm@ksu.edu.sa (M.S.S.); melrayes@ksu.edu.sa (M.M.E.R.); 2 Department of Automated Mechanical Engineering, South Ural State University, Lenin Prosp. 76, Chelyabinsk 454080, Russia; danil_u@rambler.ru; 3 Foundry Department, South Ural State University, Lenin Prosp. 76, Chelyabinsk 454080, Russia; wissenschaftler@bk.ru; 4 Department of Mechanical Design and Production, Faculty of Engineering, Zagazig University, Ash Sharqiyah 44519, Egypt; eng_mohamed_2017@yahoo.com

**Keywords:** artificial neutral network, cutting parameters, magnesium alloys, optimization, prime machining costs, surface roughness

## Abstract

Magnesium alloys are widely used in aerospace vehicles and modern cars, due to their rapid machinability at high cutting speeds. A novel Edgeworth–Pareto optimization of an artificial neural network (ANN) is presented in this paper for surface roughness (*Ra*) prediction of one component in computer numerical control (CNC) turning over minimal machining time (*T_m_*) and at prime machining costs (*C*). An ANN is built in the Matlab programming environment, based on a 4-12-3 multi-layer perceptron (MLP), to predict *Ra*, *T_m_*, and *C*, in relation to cutting speed, *v_c_*, depth of cut, *a_p_*, and feed per revolution, *f_r_*. For the first time, a profile of an AZ61 alloy workpiece after finish turning is constructed using an ANN for the range of experimental values *v_c_*, *a_p_*, and *f_r_*. The global minimum length of a three-dimensional estimation vector was defined with the following coordinates: *Ra* = 0.087 μm, *T_m_* = 0.358 min/cm^3^, *C* = $8.2973. Likewise, the corresponding finish-turning parameters were also estimated: cutting speed *v_c_* = 250 m/min, cutting depth *a_p_* = 1.0 mm, and feed per revolution *f_r_* = 0.08 mm/rev. The ANN model achieved a reliable prediction accuracy of ±1.35% for surface roughness.

## 1. Introduction

Today, many industries such as mechanical engineering, automobile manufacturing, machine-tool building, and aerospace industries, among others, all employ turning. One of the main quality parameters in finish turning [1,2,3,4,5,6,7], milling [8,9,10,11], and grinding [12,13] is surface roughness. AZ 61 magnesium alloys are widely used in industry, due to their lightweight structure [14,15]. Their basic properties mainly depend on their hexagonal mesh structure [16]. These alloys are used for many cast components in the automotive industry [17,18], such as cast magnesium engine bodies, because plastic deformation in hexagonal metal materials is of greater complexity than than in cubic metals [19]. AZ 61 magnesium alloys are also widely used in many aerospace vehicles and modern cars, in part due to the high cutting speeds of these alloys [20,21]. As well as surface quality, minimization of the use of resources is also an important objective when machining expensive materials such as AZ 61 magnesium alloys. It is essential to assure minimum machining times of unit volume and minimum surface roughness, *Ra*, simultaneously.

Plenty of research has covered the prediction of surface roughness in turning. Risbood et al. [22] established that neural networks can be used to predict surface roughness with reasonable accuracy, by using tool-holder radial vibration under acceleration as feedback. Özel and Karpat [23] used neural network modeling to predict surface roughness and tool flank wear during machining times under various cutting conditions for the finish turning of hardened AISI 52100 steel. Bajić et al. [24] examined the influence of cutting speed, feed rate, and depth of cut on surface roughness and cutting force components in longitudinal turning. Regression analysis and neural networks were applied to the surface roughness prediction model. Muthukrishnan and Davim [25] obtained a model for predicting the roughness of a machined surface using analysis of variance (ANOVA) and artificial neural network (ANN) techniques in the turning of Al/SiC-MMC workpieces. Natarajan et al. [26] described a surface roughness prediction model using Matlab-based ANN processing data on C26000 brass in turning operations under dry cutting conditions. Svalina et al. [27] analyzed the influence of cutting depth, feed rate, and the number of revolutions for ANN surface roughness prediction. Pontes et al. [28] presented a study on the applicability of radial base function (RBF) neural networks for the prediction of roughness average (*Ra*) in the turning process of SAE 52100 hardened steel, applying Taguchi orthogonal arrays as a tool to design network parameters. Hessainia et al. [29] developed surface roughness models for hard (finishing) turning of 42CrMo4 steel with an Al_2_O_3_/TiC ceramic cutting tool using the response surface methodology (RSM). Krolczyk et al. [30] identified surface integrity of the turned workpieces using fused deposition modeling (FDM). Nieslony et al. [31] presented the problem of precise turning of 55NiCrMoV6 hardened steel mould parts and demonstrated a topographic inspection of the machined surface quality. Acayaba and Escalona [32] developed a model for predicting surface roughness in the low-speed turning of AISI316 stainless steel using multiple linear regression and ANN methodologies. D’Addona and Raykar [33] studied the influence of hard turning parameters—speed, feed rate, depth of cut, and nose radius (for wipers and regular inserts)—on surface roughness. Mia and Dhar [34] obtained an ANN model for predicting average surface roughness when turning EN 24T hardened steel. Jurkovic et al. [35] compared three machine-learning methods in predicting the observed parameters of high-speed turning (surface roughness (*Ra*), cutting force (*Fc*), and tool life (*T*)). Tootooni et al. [36] used a non-contact, vision-based online measurement method for measuring surface roughness while turning the external diameter of the workpiece. Mia et al. [37] focused on developing predictive models of average surface roughness, chip-tool interface temperature, chip reduction coefficient, and average tool flank wear when turning a Ti-6Al-4V alloy. Mia et al. [38] investigated the plain turning of hardened AISI 1060 steel and examined the effect of three sustainable techniques and the traditional flood cooling system on the following machining indices: cutting temperature, surface roughness, chip characteristics, and tool wear.

Even though some studies [22,23,24,25,26,27,28,29,30,31,32,33,34,35,36,37,38] presented surface roughness prediction models, they were unable to solve the problem of establishing the cutting parameters that would yield optimal surface roughness.

Looking at the papers that describe optimal surface roughness parameters in turning, Al-Ahmari [39] developed empirical models for tool life, surface roughness, and cutting force in turning operations. Jafarian et al. [40] proposed a method for determining optimal machining parameters on the basis of three separate neural networks, both to minimize surface roughness and resultant cutting forces and to maximize tool life in the turning process. Mokhtari Homami et al. [41] used neural networks to determine optimum flank wear and surface roughness parameters when turning an Inconel 718 superalloy. Sangwan et al. [42] used ANN and the genetic algorithm (GA) to establish the optimal machining parameters as a function of minimum surface roughness, turning a Ti-6Al-4V titanium alloy. Gupta et al. [43] focused on optimization of certain process parameters of the turning operation: surface roughness, tool flank wear, and power consumption. Venkata Rao and Murthy [44] developed statistical models to investigate the effect of cutting parameters on surface roughness and root mean square of work piece vibration in the boring of AISI 316 stainless steel with physical vapor deposition (PVD)-coated carbide tools. Cutting parameters were optimized for minimum surface roughness and root mean square of work piece vibration using a multi-response optimization technique. Zerti et al. [45] solved an optimization problem of minimizing surface roughness, peripheral force, specific cutting force, and cutting power in the dry turning of AISI D3 steel. Mia et al. [46] presented optimization of cutting forces, average surface roughness, cutting temperature, and chip minimizing coefficient when turning a Ti-6Al-4V alloy under dry conditions and with high pressure coolant (HPC) simultaneously applied to the rake and the flank surfaces. Mia and Dhar [47] evaluated the effects of material hardness and high-pressure coolant jet over dry machining with respect to surface roughness and cutting temperature using a Taguchi L 36 orthogonal array.

However, studies with the objective of establishing the optimal cutting modes are limited [39,40,41,42,43,44,45,46,47] in that they only take into account surface roughness and not its interconnection with processing performance and unit-volume machining time, which is unacceptable when processing such expensive materials such as AZ 61 magnesium alloys.

Following the above, the papers that describe optimal turning parameters using multi-objective optimization may be considered [48,49,50,51,52]. Basak et al. [48] described two types of Pareto optimization: minimization of production time and minimization of the cost of machining. Surface roughness was considered to be a limitation. Karpat and Özel [49] used neural networks and multi-objective Pareto optimization to establish machining parameters in longitudinal turning of hardened AISI H13 steel. The optimization criteria were defined as follows: minimize surface roughness values and maximize productivity, maximize tool life, and material removal rate, and minimize machining induced stresses on the surface and, likewise, surface roughness. Raykar et al. [50] used grey relational analysis (GRA) to investigate the high-speed turning of Al 7075 high-strength aluminium alloy. As a result of GRA-based multipurpose optimization, the optimum conditions were established for the given surface roughness, energy consumption, material removal rate, and cutting time while turning. Yue et al. [51] used multi-objective Pareto optimization to establish the relation between surface roughness, thickness of plastic deformation, and cutting conditions in the hard turning of Cr12MoV die steel. Abbas et al. [52] obtained a Pareto frontier for *Ra* and *T_m_* of the finished workpiece from high-strength steel using the ANN model that was later used to determine the optimum finishing cutting conditions. There is, therefore, little research dedicated to multi-objective optimization in turning. The most efficient approach to solving such problems is Pareto optimization. However, studies [48,49,50,51,52] are not concerned with multiobjective optimization of machining AZ61 magnesium alloys. Taking into account the high cost of this material, it is necessary to ensure the design roughness value of the machined surface and the minimum processing time of the material volume at minimal processing costs.

The objective of this study is, therefore, to establish the turning conditions of AZ61 magnesium alloys that provide the minimum unit-volume machining time, *T_m_*, the minimum surface roughness, *Ra*, and the minimum cost of machining one part, *C*.

## 2. Experiment

Magnesium alloy AZ 61 contains aluminum (nominally 6%), zinc (nominally 1%), and other trace elements. Table 1 summarizes the chemical composition of AZ 61.

**Figure 1:**
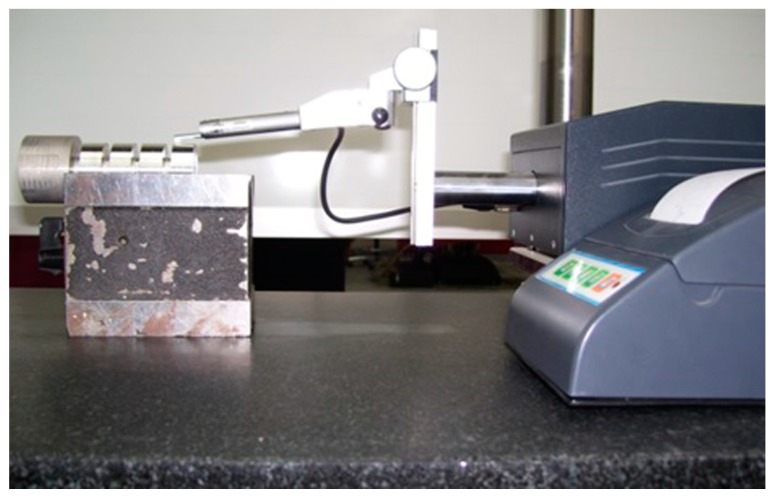
Test rig for measuring surface roughness.

The CNC lathe used to conduct the experiments was an EMCO Concept Turn 45 (Emco, Salzburg, Austria) equipped with Sinumeric 840-D, a SVJCL2020K16 tool holder, and a VCGT 160404 FN-ALU insert. The cutting edge angle (*k_r_*), back angle (α), and nose radius (*r_o_*) were set at 35°, 5°, and 0.4 mm, respectively. Workpiece length (*L*), workpiece diameter (*D*) and tolerance (*l*_1_) were set at 20, 40, and 2 mm, respectively. Experiments were carried out under wet cutting conditions. A TESA Rugosurf 90-G surface roughness tester (TESA, Bugnon, Switzerland) was used. Figure 1 illustrates the test rig used to measure surface roughness. 

The test plan was implemented through 64 turning runs divided into 16 groups. For each set of four groups, one common cutting speed, *v_c_*, was used (100, 150, 200, and 250 m/min). Each set of experiments was machined using four different depths of cut *a_p_* (0.25, 0.50, 0.750, and 1.00 mm). Each depth of cut was processed using four levels of feed rate *f_r_* (0.04, 0.08, 0.12, and 0.16 mm/rev). The surface roughness values for the different cutting conditions are presented in Table 2.

Table 3, below, summarizes the basic economic parameters for optimizing the turning of an AZ61 aluminum alloy workpiece.

## 3. System Adaptation Procedure

The procedure for system adaptation is described as follows:

Step one: Posing a multiobjective optimization problem, i.e., establishing the criteria, limitations, and boundary conditions.

Step two: Establishing a relationship between the parameters of the cutting tool–workpiece system, i.e., build and train an ANN.

Step three: Graphically interpreting the surfaces of normalized three-dimensional space, determine the states of the system in which the values of each particular indicator cannot be improved without aggravating others, i.e., the Pareto frontier.

Step four. Establishing the optimum turning conditions for the workpiece, i.e., to adapt the cutting tool–workpiece system to the given conditions.

Before we begin the procedure, the nomenclature that we will use is introduced. DM—decision maker; m—a number of criteria; I = {1, 2, …, *m*}—a set of criteria numbers; X—a set of possible decisions; *f* = (*f*_1_, *f*_2_, …, *f_m_*)—vector-valued criterion; Y = *f*(X)—a set of possible vectors (estimates); R^m^—Euclidean space of m-dimensional vectors with real components; > _X_—preference relation of DM specified in the set X; > _Y_—preference relation of DM, induced on the set with > _X_ and specified in the set Y; >—relation > _Y_ continued in the entire space R^m^; Sel X—a set of selected decisions; Sel Y—a set of selected vectors (estimates); Ndom X—a set of non-dominated decisions; Ndom Y—a set of non-dominated vectors (estimates); P_f_ (X)—a set of Pareto optimal decisions; P(Y)—a set of Pareto optimal vectors (Pareto optimal estimates).

## 4. Formulation of an Optimization Problem

This investigation of machining operations has the objective of resolving the following optimization problem criteria: *f*_1_—surface roughness (*Ra*, μm) and *f*_2_—unit-volume machining time volume in one cutting tool pass (*T_m_*, min/cm^3^); and, *f*_3_—the cost price of processing one component part (*C*, $), i.e., *m* = 3. Relatively, a set of possible Y estimates in the two-dimensional space, *R*^3^, is formed with vectors *f* = (*f*_1_, *f*_2_, *f*_3_). The search is performed for a set of estimates with the minimum length of vector *f*, which is a vector from the coordinate origin to a point on the estimate surface. The criteria are presented in a normalized dimensionless form with index 1 assigned to the maximum actual numbers.

As the adaptation of the system takes place, the parameters are varied in accordance with the following experimental table (see Table 2): *x*_1_ = [100 ÷ 250]; cutting speed, *v_c_*, m/min; *x*_2_ = [0.25 ÷ 1.0]; depth of cut, *a_p_*, mm; *x*_3_ = [0.04 ÷ 0.16]; and, feed rate, *f_r_*, mm/rev.

We will evaluate the state of the system based on four criteria (Table 4, Table 5, Table 6 and Table 7). The first criterion is surface roughness *Ra* (µm), and dimensionless surface roughness Ra*(f_1_). The second criterion is unit-volume machining time, *T_m_* (min/cm^3^), and unit-volume dimensionless machining time T_m_*(*f_2_*). The third criterion is the cost price of processing one component part, *C* ($), or the dimensionless cost price of processing one component part, C* (*f*_3_). The fourth criterion is the dimensionless vector of estimates in a three-dimensional normalized space, *f*.

The values of the first criterion are taken from the experimental table and the rest are calculated on the basis of Formulas (1)–(6):

*T_m_* = 1/(1000 × *v_c_* × *a_p_* × *f_r_*); (1)

*С_i_* = (*C_Mh_* × *T^/^*) + (*C_Toolmin_* × *T^/^*) + *C_w_*, where is Machining Time in Turning *T^/^* = (*L* + *l*_1_)/(*n* × *f_r_*), where

*n* = (1000·*v_c_*)/(3.141·*D*); (2)

Ra* = *Ra_i_*/*Ra*_max_; (3)

T_m_* = *T_m i_*/*T_m_*_max_; (4)

C* = *C_i_*/*C_i_*_max_; (5)

(6) $$f=\sqrt{f_{1}^{2}+f_{2}^{2}+f_{3}^{2}}+\sqrt{R_{a}^{*2}+T_{m}^{*2}+C^{*2}},$$

where, *Ra_i_* is surface roughness for the current combination of X... and *f_r_*; *Ra_max_* is the maximum surface roughness value of all the *v_c_, a_p_*, and *f_r_* combinations; *T_m i_* is the unit-volume machining time for the current values of *v_c_*, *a_p_*, and *f_r_*; *T_m max_* is the maximum unit-volume machining time of all the *v_c_*, *a_p_*, and *f_r_* combinations; *С_i_* is the cost price of processing one part for a given combination of *v_c_*, *a_p_*, and *f_r_*; *C_i max_*—the maximum value.

The optimum search procedure involves a non-negative set of vector estimates, and eliminates the variation of parameter values below zero. The boundary condition is, therefore, that all the variables in this model are non-negative.

Now that the optimization problem is formulated, we shall build and train the neural network that should become the functional operator of the three variables *f*(*f*_1_, *f*_2_, *f*_3_) and *f*(*f*_1_, *f*_2_, *f*_3_), as well as the functional Q to the plane *f*(*f*_1_, *f*_2_, *f*_3_). The ANN complex was constructed using the Skif AURORA-SUSU supercomputer cluster (South Ural State University, Chelyabinsk, Russia) [53].

## 5. Building a Neural Network Model

Matlab today outperforms other well-known software packages—Maple, Mathematica, and Mathcad—in terms of fundamental quality and versatile numerical calculations. Neural networks can be designed, modeled, and trained easily with the Matlab neural network toolbox. A clear advantage of Matlab is its programming language that can be used to write algorithms and programmes. Many tasks can be achieved with its versatile language, including: data collection, analysis, and structuring, adding to algorithms, system modeling, debugging, object-oriented programming, and graphical user interface development. Matlab applications may also be converted to either C or C++ code.

The programming environment Matlab R2010b, a parallel version of Matlab, was selected in this study. A multi-layer perceptron (MLP) using the Levenberg–Marquardt algorithm was used to train the controlled feedforward neural network. Sigmoid neurons in a hidden layer and output neurons in a linear layer form the network structure; the best structure for multidimensional mapping problems.

The network was trained with only the maximums of the normalized values. Training efficiency was improved with these values within the [0, 1] range.

Improvements to the generalization performance of the network corrected overfitting through the use of a pair of data sets: a training set that, if undesired events took place, updated weights and offsets and a validation set that could stop the training.

The final network configuration (total neurons in the hidden layer) was defined by the lowest mean squared error of the validation set.

**Figure 2:**
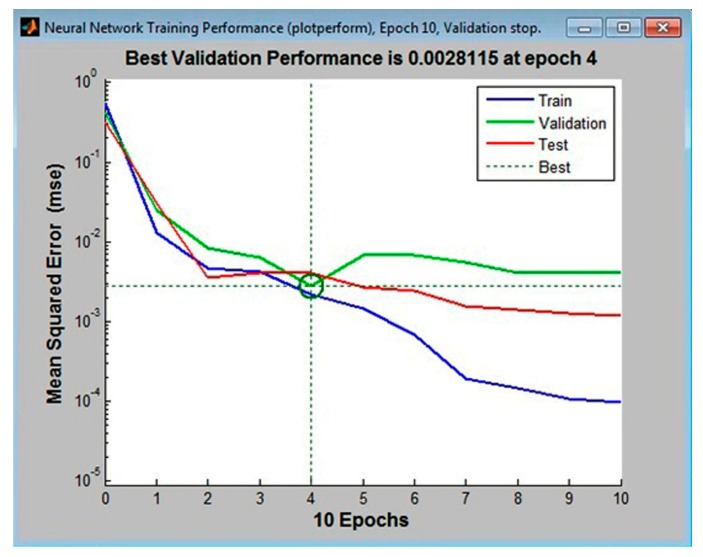
The lowest mean squared error for the validationset in the multi-layer perceptron (MLP) 3-11-4 configuration (calculated in Matlab).

**Figure 3:**
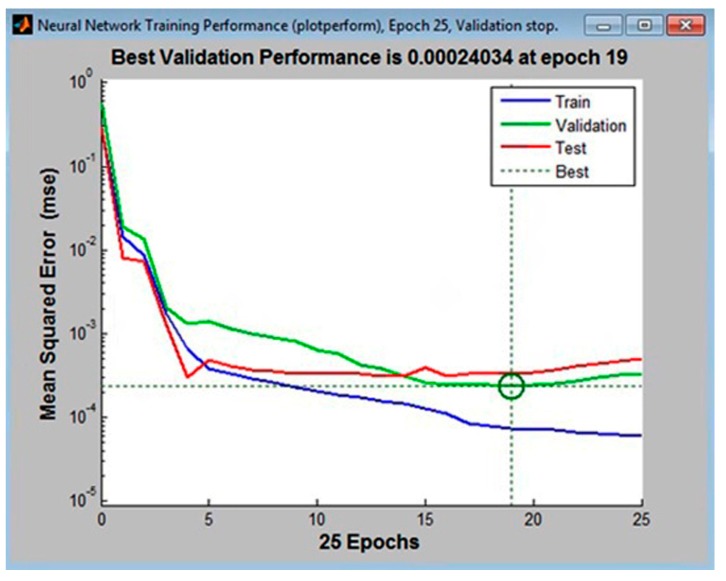
The lowest mean squared error for the validationset in the MLP 3-12-4 configuration (calculated in Matlab).

**Figure 4:**
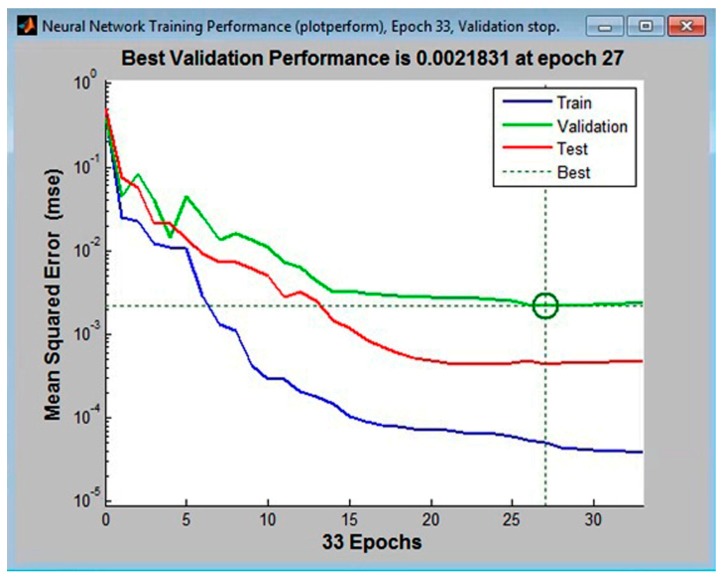
The lowest mean squared error for the validation set in the MLP 3-13-4 configuration (calculated in Matlab).

A hidden layer with 11, 12, and 13 neurons, with 10% of the tabular data assigned to the validation set, was first used to train the multilayer perceptrons. The following configurations had the lowest error values: MLP 3-11-4, the MLP 3-12-4, and MLP 3-14-4. These are shown in Figure 2, Figure 3 and Figure 4, respectively.

**Figure 5:**
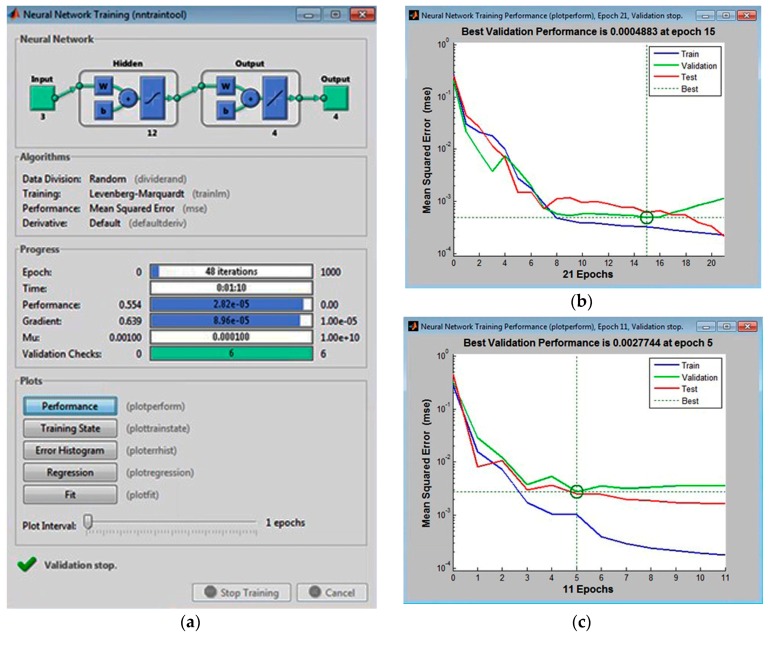
The lowest mean squared error in generalizing experimental data in MLP 3-12-4 (**a**) with various validation sets: (**b**) 5%; (**c**) 15% (calculated in Matlab).

Analysis of the graphical functions presented in Figure 2, Figure 3 and Figure 4 showed that the MLP 3-12-4 configuration had the lowest error, 0.002%, in the validation set. The coefficient of determination of the model with respect to criterion *f* was 0.986, which reflects its high accuracy in predicting surface roughness (±1.35%). The same model appeared to be the best at generalization performance in the cases of assigning 5% or 15% in the validation set of tabular data, Figure 5. In the case of assigning 5% of the training set, the error was 0.004% (see Figure 5b), and in the case of assigning 15%, it was 0.027% (see Figure 5c).

## 6. Graphical Representation of the Surface of Vector Estimates (D)

The first step was to build the surface of vector estimates, D. The MLP 3-12-4 model and the experimental values of *x*_1_, *x*_2_, and *x*_3_ were used to calculate *f*_1_, *f*_2_ and *f*_3_.

**Figure 6:**
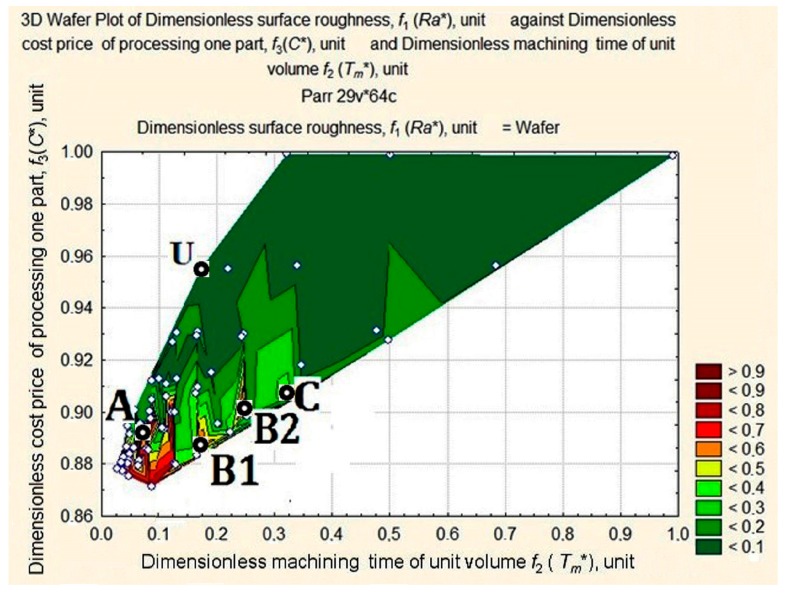
Wafer map of workpiece Ra* after machining with respect to changes in values of T_m_* and C*.

A projection (a wafer map of Ra* values) of non-linear surface D was plotted on the plane *f*_2_ *f*_3_ (Tm* С*) and is shown Figure 6.

An analysis of the wafer map of Ra* shows elements, forms, and complexes that can all be located. We can clearly see the apex A in the area of minimal values of C* and T_m_* and the quasi-horizontal plane with a dent U in the opposite area of C* and T_m_* values. We can also clearly see apexes B1, B2, and C with slopes towards the dent.

**Figure 7:**
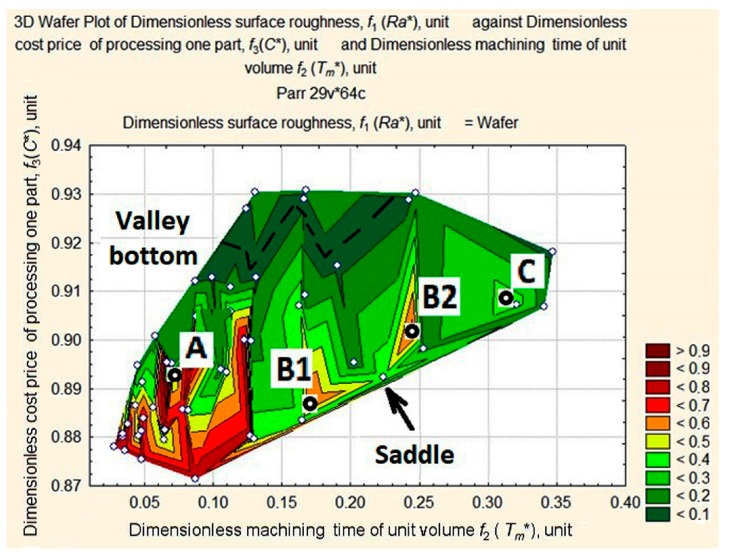
Apexes of the wafer map of Ra* values of the machined workpiece depending on the change in values of T_m_* and C*.

A closer look at the apexes on the map can be seen in detail in Figure 7.

In Figure 7, we can see that the highest apex А (0.0667; 0.8953; 0.9868) appears as a ridge, В1 (0.1712; 0.8873; 0.5861) and В2 (0.2481; 0.9014; 0.6630) as mountains, and the lowest apex С (0.3154; 0.9082; 0.3721) appears to be a hill. B1 and B2 are similar in shape and form something like a mountain system with a saddle. A thalweg is plotted in the valley hollow.

## 7. Establishment of a Pareto Frontier

**Figure 8:**
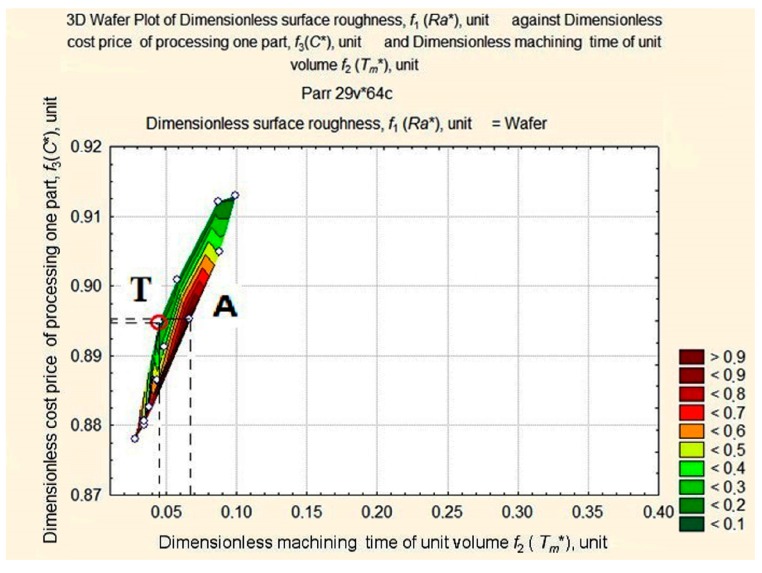
Surface projection of Ra* values depending on the change in the values of T_m_* and C* at fixed depth of cut, *a_p_* = 1 mm.

**Figure 9:**
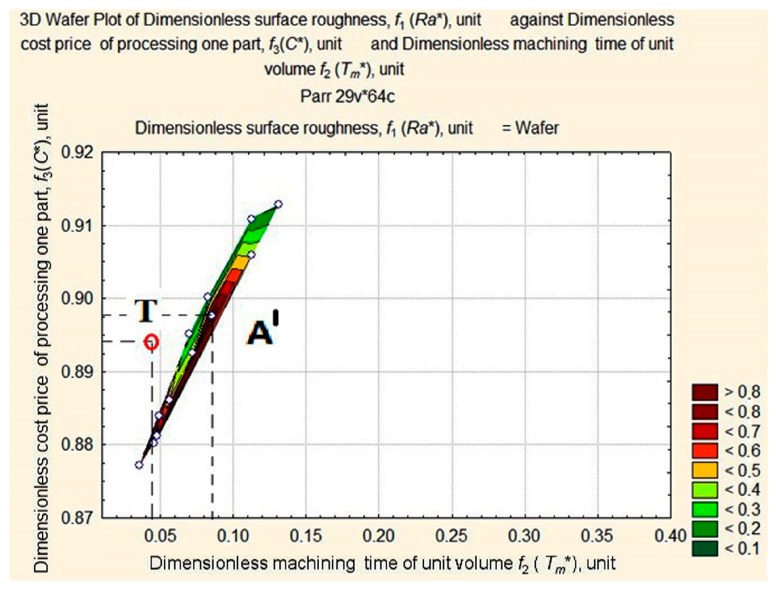
Surface projection of Ra* values dependent on the change in the values of T_m_* and C* at a fixed depth of cut, *a_p_* = 0.75 mm.

**Figure 10:**
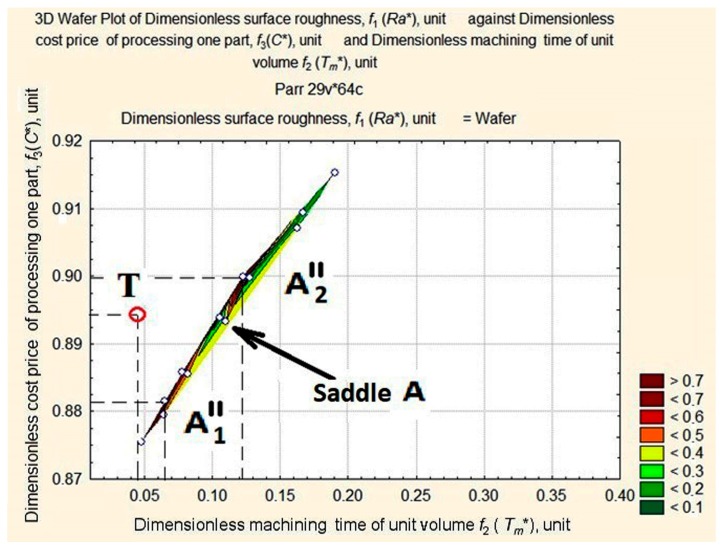
Surface projection of Ra* values depending on the change in the values of T_m_* and C* at a fixed depth of cut, *a_p_* = 0.5 mm.

**Figure 11:**
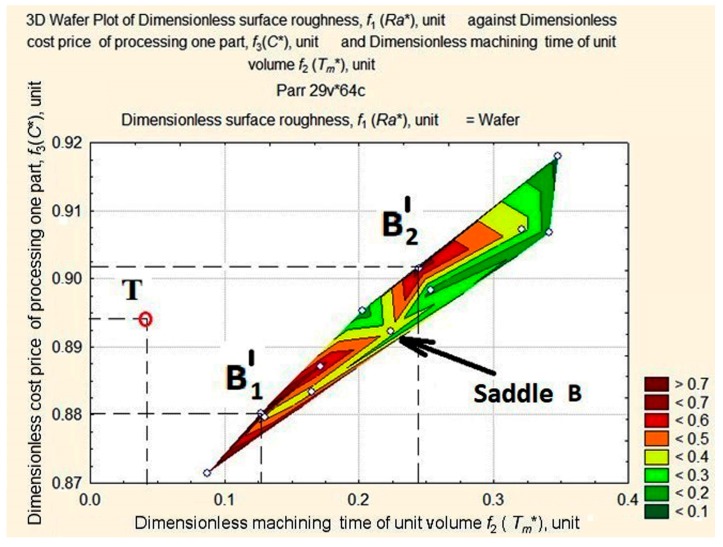
Surface projection of Ra* values depending on the change in the values of T_m_* and *C* at a fixed depth of cut, *a_p_* = 0.25 mm.

The target function is represented by the length of the vector in a normalized space and connects the origin of the coordinates with the point of the three-dimensional surface of estimates. Our aim is to identify the smallest of its lengths at the foot of the Ra* ridge in the area with the smallest values of C* and T_m_* (see Figure 8). For this purpose, we shall consider surface projection estimates at fixed depths of cut—*a_p_* = 1 mm, *a_p_* = 0.75 mm, *a_p_* = 0.5 mm, and *a_p_* = 0.25 mm (Figure 8, Figure 9, Figure 10 and Figure 11).

The above figures (see Figure 8, Figure 9, Figure 10 and Figure 11) illustrate how the area of maximum values of dimensionless roughness is transformed as the depth of cut decreases from *a_p_* = 1.0 to *a_p_* = 0.25 mm. It changes from a merged apex A and A’ to a maximum with two peaks in A1″, A″ with saddle A and B1′, and B2′ with saddle B. The largest displacement of the peaks occurs at the T_m_* coordinate with the distance between them increasing. The ridge (see Figure 10) is formed by peaks А, А′, А1″ and А2″, the mountain system, by peaks В1′and В2′, and the hill, with the long slope of peak В2′ and the dent, U.

As a result of analyzing an MLP 3-12-4 neural network model with the coefficient of determination, R^2^ = 0.986 (accuracy ±1.35%), a pattern was revealed for the AZ61 alloy. When the other two parameters were fixed and the cutting speed, v_c_*, was increased by 0.1 units, the values of Ra*, T_m_*, C*, and f* decreased by 0.001 units, 0.007 units, 0.003 units, and 0.005 units, respectively. When the other two parameters were fixed and the depth of cut, a_p_*, was increased by 0.1 units, the values of Ra*, T_m_*, C*, and f* decreased by 0.007 units, 0.010 units, 0.0001 units, and 0.005 units, respectively. When the other two parameters were fixed and the feed rate, f_r_*, was increased by 0.1 units, T_m_*, and C* decreased by 0.004 and 0.006 units, respectively, while Ra* and f* increased by 0.103 and 0.015 units, respectively. Compared to *v_c_*, surface roughness (*Ra*) was 7.3 times more affected by *a_p_* and 102.9 times more affected by *f_r_*; machining time (*T_m_*) was 1.5 times less affected by *a_p_* and 1.5 times more affected by fr; cost of production (*C*) was 26 times less affected by *a_p_* and 1.9 times more affected by *f_r_*; the integrated optimization criterion (*f*) was 1.04 times more affected by *a_p_* and 2.9 times more affected by *f_r_*. Hence, we should look for the optimal cutting conditions at the maximum cutting speed and depth of cut and the minimum feed rates.

The optimum must be located at the foot of the ridge in the area of high-speed turning conditions that are most likely to lead to maximum tool wear. According to Figure 8, Figure 9, Figure 10 and Figure 11, we limit ourselves to the cutting conditions at the depth of cut *a_p_* = 1 mm, as in this case all values of Ra* are located near the minimum vector estimation F (0.0449; 0.8948; 0.1253), which is marked by point T.

**Figure 12:**
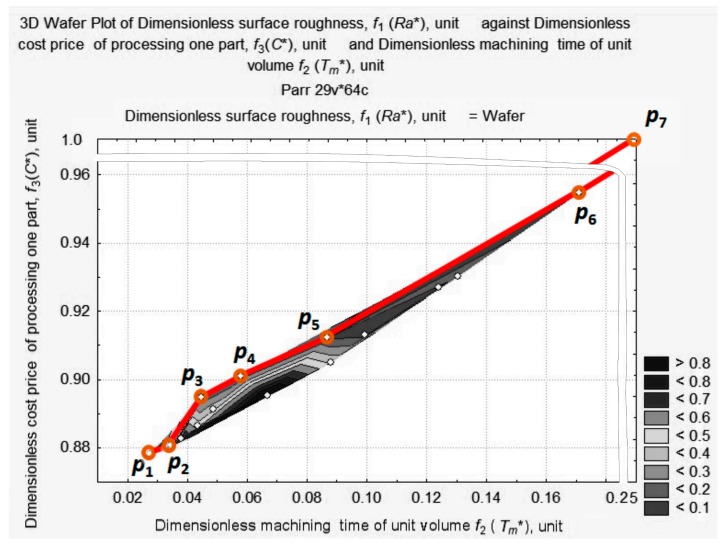
Pareto frontier and seven reference points: p_1_ (0.0281; 0.8782; 0.8135); p_2_ (0.0343; 0.8824; 0.8273); p_3_ (0.0449; 0.8948; 0.1253); p_4_ (0.0561; 0.9012; 0.1072); p_5_ (0.0860; 0.9123; 0.0982); p_6_ (0.1710; 0.9547; 0.0514); p_7_ (0.2500; 1.0000; 0.7903).

At this depth of cut, *a_p_* = 1 mm, four graphic dependencies, Ra* = *f*(C*, T_m_*), were constructed, corresponding to the fixed *v_c_* = 250 m/min, *v_c_* = 200 m/min, *v_c_* = 150 m/min, *v_c_* = 100 m/min, and variable, *f_r_*, value. After matching the obtained curves with the projection (see Figure 8), we obtained the seven reference points of the Pareto frontier. They are shown in Figure 12: p_1_ (0.0281; 0.8782; 0.8135); p_2_ (0.0343; 0.8824; 0.8273); p_3_ (0.0449; 0.8948; 0.1253); p_4_ (0.0561; 0.9012; 0.1072); p_5_ (0.0860; 0.9123; 0.0982); p_6_ (0.1710; 0.9547; 0.0514); p_7_ (0.2500; 1.0000; 0.7903).

Six sections are visible on the Pareto frontier.

Section 1 between point p_1_ and point p_2_, corresponds to *v_c_ =* 250–200 m/min, *a_p_* = 1.00 mm, *f_r_* = 0.16 mm/rev. Section 2 between point p_2_ and point p_3_ corresponds to *v_c_* = 250 m/min, *a_p_* = 1.00 mm, *f_r_* = 0.16–0.08 mm/rev. Section 3 between point p_3_ and point p_4_ corresponds to *v_c_* = 250–200 m/min, *a_p_* = 1.00 mm, *f_r_* = 0.08 mm/rev. Section 4 between points p_4_ and p_5_ corresponds to *v_c_* = 200–150 m/min, *a_p_* = 1.00 mm, *f_r_* = 0.08 mm/rev. Section 5 between point p_5_ and point p_6_ corresponds to *v_c_* = 250 m/min, *a_p_* = 1.00 mm, *f_r_* = 0.12–0.16 mm/rev. Section 6 between point p_6_ and point p_7_ corresponds to *v_c_* = 150–100 m/min, *a_p_* = 1.00 mm, *f_r_* = 0.16 mm/rev. p_3_ and p_6_ are special points on the Pareto curve. These points correspond to absolute minimums; p_3_ is the absolute minimum of the length of vector *f*; and p_6_ is the absolute minimum of surface roughness.

## 8. The Optimum Settings

**Figure 13:**
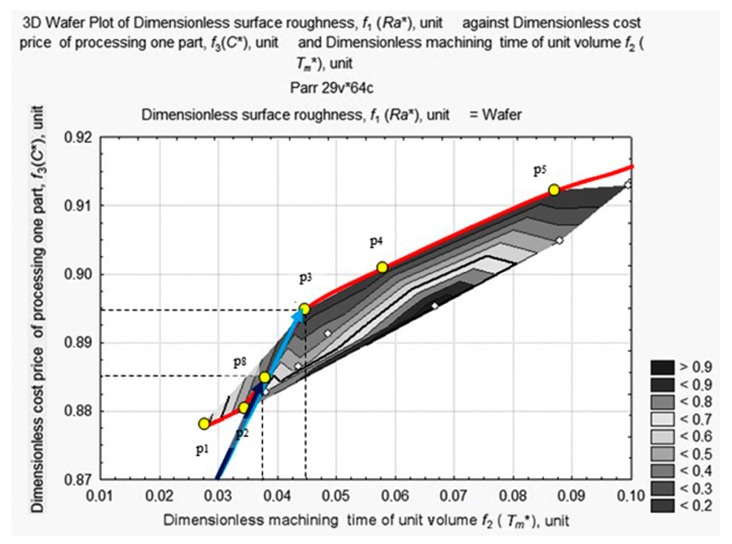
Global and local minimums on the Pareto curve.

Finally, the procedure to adapt the system requires us to establish the optimum settings. The Pareto optimal decisions have to be narrowed down to a set of Pareto non-dominated decisions. Expert assessment defined the lesser importance of the dimensionless criterion of surface roughness, compared to the unit-volume machining time, Tm*, and the processing cost price, *C_i_*. In consequence, all vectors located above the blue vector that has the lowest *f* vector, plotted on the *f*_3_ *f*_2_ plane, at an angle of 7.89°, represent the Pareto non-dominated estimates (Figure 13). Point 3 on the Pareto curve coincides with the end point of this vector that was the global minimum in the case of unconditional optimization with the ranking *f*_1_:*f*_2_:*f*_3_ = 1.0:19.9:2.7. Using the real coordinates, the global minimum corresponds to *T_m_* = 0.358 min/cm^3^, *С* = $8.2973, *R_a_ =* 0.087 μm, *v_c_* = 250 rpm, *a_p_* = 1.0 mm, and *f_r_* = 0.08 m/min.

Further restrictions were imposed in the form of design documentation requirements and the minimum acceptable surface roughness value was defined at 0.800 μm, which is point p_8_ on the Pareto frontier curve, having the coordinates (0.0372, 0.8851, 0.300) in Figure 13. In this case (see Figure 13, blue estimates vector), the optimization criteria has a valid relation of importance that is–Ra*/T_m_*/С* = 1.0/27.6/9.3, and the valid preference for points eight and three becomes y_8_ > _Y_ y_3_ with an induced preference of x_8_ > _X_ x_3_.

Therefore, the selected estimates as a set, Sel Y, was restricted to the blue vector, the actual end coordinates of which were 0.0372, 0.8851, 0.300, and the set of selected decisions, Sel X, was restricted to the three-dimensional vector of the optimum cutting parameters (*v_c_ =* 248 rpm, *a_p_ =* 1.0 mm, *f_r_* = 0.10 mm/min).

**Figure 14:**
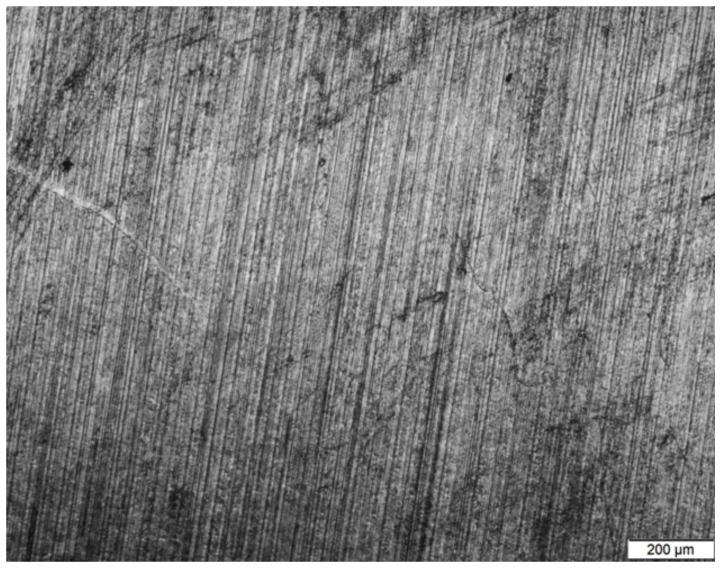
Optical microscopy results for the optimal machining parameters.

**Figure 15:**
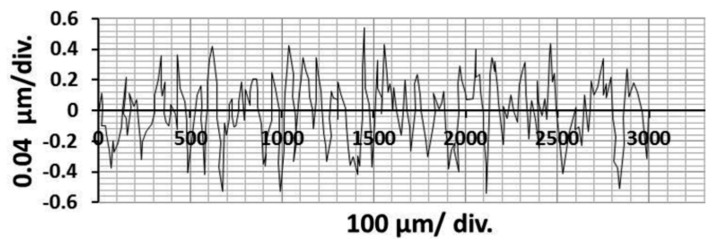
Profile of surface roughness graph from the surface roughness tester for the optimal machining parameters.

The optical microscopy results and the profile of surface roughness graphs for the global optimum (cutting speed *v_c_* = 250 m/min, depth of cut *a_p_* = 1.0 mm, and feed rate *f_r_* = 0.08 mm/rev) are presented in Figure 14 and Figure 15.

All in all, note that the Pareto curve, the correct values of its points, and the vector coordinates were all automatically calculated with a customized function in Matlab based on a neural network model. Implementation of the strategy in Matlab permits rapid calculation of the cutting tool–workpiece system and its local optimums in computer-aided production systems over the entire range of speeds and cutting depths.

## 9. Conclusions

(1) For the first time in the turning of magnesium alloy, the Edgeworth–Pareto methodology has been used for adapting the cutting tool–workpiece system to the state of the minimal value of the three-dimensional estimates of vector *f* in a normalized space: *f*_1_ *f*_2_ *f*_3_ using an artificial intelligence-based model.
(2) An artificial neural network has been created in the Matlab programming environment based on an MLP 4-12-3 multi-layer perceptron that predicts the values of *f*_1_, *f*_2_, *f*_3_, *f* in the finishing turning of the AZ61 magnesium alloy workpiece with a width of X mm, a length of X mm, and a height of X mm, at a cutting speed of 100–250 m/min, a depth of cut from 0.25 to 1.0 mm, and a feed rate of 50–150 mm/rev with an accuracy of ±1.35%.
(3) According to the neural network model for the AZ61 alloy in finish turning, the value of the integrated optimization criterion, *f*, has mainly been influenced by feed rate, *f_r_*. Vector *f* is 2.9 times more influenced by feed rate than by cutting speed and depth of cut. Increasing the feed rate led to an increase in *f*, and increasing *v_c_* and *a_p_* led to a decrease in *f*.
(4) For the first time, an AZ61 magnesium alloy workpiece wafer plot of surface roughness after finishing turning has been generated at cutting speeds of 100–250 m/min, at a depth of cut from 0.25–1.0 mm, and at a feed rate of 50–150 mm/rev.
(5) The global optimum in the finish turning of the alloy workpiece has been set as follows: the minimum length of 3D vector estimates with the coordinates *Ra* = 0.087 μm, *T_m_* = 0.358 min/cm^3^, and *С* = $8.2973 corresponded to the following optimum conditions of finishing turning: cutting speed *v_c_* = 250 m/min, depth of cut *a_p_* =1.0 mm, and feed rate *f_r_* = 0.08 mm/rev.
(6) Automated calculation with the Industry 4.0 Framework has been performed in the Matlab environment, to define the optimal turning conditions for magnesium alloy workpieces as products of intelligent computer-aided manufacturing systems.

## Figures and Tables

**Table 1:** Chemical composition of AZ 61 magnesium alloy.

Element	Aluminum	Zinc	Copper	Silicon	Iron	Nickel	Magnesium
Mass %	6	0.90	0.02	0.008	0.007	0.003	Balance

**Table 2:** Surface roughness values under different cutting conditions.

Cutting Speed: *v_c_*, (m/min)	Feed: *f_r_*, (mm/rev)	Surface Roughness: *Ra* (µm)			
		Depth of Cut: *a_p_*, (mm)			
		0.25	0.5	0.75	1.0
100	0.0400	0.1730	0.1660	0.1500	0.1290
100	0.0800	0.3880	0.3610	0.3530	0.4400
100	0.1200	0.8720	0.9520	1.0470	1.0200
100	0.1600	1.6780	2.1040	2.1790	2.6290
150	0.0400	0.1460	0.1320	0.1160	0.1890
150	0.0800	0.3440	0.3480	0.3150	0.4130
150	0.1200	0.9310	1.0540	0.9840	0.9990
150	0.1600	1.6370	1.7640	1.7020	1.8840
200	0.0400	0.1820	0.1800	0.2040	0.1500
200	0.0800	0.3670	0.3860	0.3970	0.3550
200	0.1200	0.8450	1.0240	1.0340	1.2140
200	0.1600	1.9760	1.9220	1.9350	2.0140
250	0.0400	0.1230	0.1830	0.1370	0.2240
250	0.0800	0.3590	0.3890	0.3580	0.3250
250	0.1200	0.9370	0.9680	0.9500	1.0000
250	0.1600	2.0880	1.9540	2.0170	1.8930

**Table 3:** Summary of basic economic parameters.

Mater.	Cost of Machining/Hour (SR 400), *CMh*: $	Cost of Tool Holder, *CToolh*: $	Tool Holder Life: *LTToolh* min	Cost of Insert, *CIn*: $	Setup Insert: *k*	Unit Cost of Work-Piece: *Cw*: $	Tool Life: *T* Min	Cost of Tool Minute: *CToolmin*, $ *CToolmin* = (*CIn*/(*T*×*k*)) + (*CToolhLTToolh*)
AZ61	106	85	5 Year × 365 Day × 24 h × 60 min = 2,628,000	10	2	8	60	0.083

**Table 4:** Optimization criteria for the variable machining parameters at a fixed depth of cut—*a_p_* = 0.25 mm.

Variable Parameters			Optimization Criteria							
*x*_1_ Cutting Speed: *v_c_*, (m/min)	*x*_2_ Depth of Cut: *a_p_*, (mm)	*x*_3_ Feed: *f_r_*, (mm/rev)	Surface Roughness: *Ra* (µm)	Dimensionless Surface Roughness: *f*_1_ (Ra*), u	Unit Volume Machining Time: *T_m_* (min/cm^3^)	Dimensionless Volume Machining Time: *f*_2_ (T_m_*), u	Unit cost Price of Processing One Part: *C*, ($)	Dimension-less Cost Price of Processing One Part: *f*_3_ (C*), u	Length of Estimates Vector: *f*, u	Length of Estimates Vector: f*, u
0.4	0.25	0.25	0.1730	0.0660	1.0000	1.0000	9.2729	1.0000	1.4160	1.0000
0.4	0.25	0.5	0.3880	0.1480	0.5000	0.5000	8.6374	0.9310	1.1780	0.8319
0.4	0.25	0.75	0.8720	0.3320	0.3333	0.3330	8.4237	0.9080	1.1260	0.7952
0.4	0.25	1.0	1.6780	0.6380	0.2500	0.2500	8.3187	0.8970	1.2090	0.8538
0.6	0.25	0.25	0.1460	0.0560	0.6667	0.6670	8.8492	0.9540	1.2570	0.8877
0.6	0.25	0.5	0.3440	0.1310	0.3333	0.3330	8.4237	0.9080	1.0840	0.7655
0.6	0.25	0.75	0.9310	0.3540	0.2222	0.2220	8.2837	0.8930	1.0700	0.7556
0.6	0.25	1.0	1.6370	0.6230	0.1667	0.1670	8.2118	0.8860	1.1580	0.8178
0.8	0.25	0.25	0.1820	0.0690	0.5000	0.5000	8.6374	0.9310	1.1710	0.8270
0.8	0.25	0.5	0.3670	0.1400	0.2500	0.2500	8.3187	0.8970	1.0360	0.7316
0.8	0.25	0.75	0.8450	0.3210	0.1667	0.1670	8.2118	0.8860	1.0270	0.7253
0.8	0.25	1.0	1.9760	0.7520	0.1250	0.1250	8.1584	0.8800	1.2100	0.8545
1.0	0.25	0.25	0.1230	0.0470	0.4000	0.4000	8.5084	0.9180	1.1160	0.7881
1.0	0.25	0.5	0.3590	0.1370	0.2000	0.2000	8.2542	0.8900	1.0050	0.7097
1.0	0.25	0.75	0.9370	0.3560	0.1333	0.1330	8.1695	0.8810	1.0180	0.7189
1.0	0.25	1.0	2.0880	0.7940	0.1000	0.1000	8.1271	0.8760	1.2240	0.8644

**Table 5:** Optimization criteria values for the variable parameters of machining at a fixed depth of cut—*a_p_* = 0.5 mm.

Variable Parameters			Optimization Criteria							
*x*_1_ Cutting Speed: *v_c_*, (m/min)	*x*_2_ Depth of Cut: *a_p_*, (mm)	*x*_3_ Feed: *f_r_*, (mm/rev)	Surface Roughness: *Ra* (µm)	Dimensionless Surface Roughness: *f*_1_ (Ra*), u	Unit Volume Machining Time: *T_m_* (min/cm^3^)	Dimensionless Volume Machining Time: *f*_2_ (T_m_*), u	Unit Cost Price of Processing One Part: *C*, ($)	Dimensionless Cost Price of Processing One Part: *f*_3_ (C*), u	Length of Estimates Vector: *f*, u	Length of Estimates Vector: f*, u
0.4	0.5	0.25	0.1660	0.0630	0.5000	0.5000	9.2729	1.0000	1.2260	0.8658
0.4	0.5	0.5	0.3610	0.1370	0.2500	0.2500	8.6374	0.9310	1.0660	0.7528
0.4	0.5	0.75	0.9520	0.3620	0.1667	0.1670	8.4237	0.9080	1.0590	0.7479
0.4	0.5	1.0	2.1040	0.8000	0.1250	0.1250	8.3187	0.8970	1.2530	0.8849
0.6	0.5	0.25	0.1320	0.0500	0.3333	0.3330	8.8492	0.9540	1.1160	0.7881
0.6	0.5	0.5	0.3480	0.1320	0.1667	0.1670	8.4237	0.9080	1.0040	0.7090
0.6	0.5	0.75	1.0540	0.4010	0.1111	0.1110	8.2837	0.8930	1.0340	0.7302
0.6	0.5	1.0	1.7640	0.6710	0.0833	0.0830	8.2118	0.8860	1.1480	0.8107
0.8	0.5	0.25	0.1800	0.0680	0.2500	0.2500	8.6374	0.9310	1.0590	0.7479
0.8	0.5	0.5	0.3860	0.1470	0.1250	0.1250	8.3187	0.8970	0.9750	0.6886
0.8	0.5	0.75	1.0240	0.3900	0.0833	0.0830	8.2118	0.8860	1.0100	0.7133
0.8	0.5	1.0	1.9220	0.7310	0.0625	0.0630	8.1584	0.8800	1.1710	0.8270
1.0	0.5	0.25	0.1830	0.0700	0.2000	0.2000	8.5084	0.9180	1.0240	0.7232
1.0	0.5	0.5	0.3890	0.1480	0.1000	0.1000	8.2542	0.8900	0.9560	0.6751
1.0	0.5	0.75	0.9680	0.3680	0.0667	0.0670	8.1695	0.8810	0.9890	0.6984
1.0	0.5	1.0	1.9540	0.7430	0.0500	0.0500	8.1271	0.8760	1.1700	0.8263

**Table 6:** The values of optimization criteria for the variable parameters of machining at fixed depth of cut—*a_p_* = 0.75 mm.

Variable Parameters			Optimization Criteria							
*x*_1_ Cutting Speed: *v_c_*, (m/min)	*x*_2_ Depth of Cut: *a_p_*, (mm)	*x*_3_ Feed: *f_r_*, (mm/rev)	Surface Roughness: *Ra* (µm)	Dimensionless Surface Roughness: *f*_1_ (Ra*), u	Unit Volume Machining Time: *T_m_* (min/cm^3^)	Dimensionless Volume Machining Time: *f*_2_ (T_m_*), u	Unit Cost Price of Processing One Part: *C*, ($)	Dimensionless Cost Price of Processing One Part: *f*_3_ (C*), u	Length of Estimates Vector: *f*, u	Length of Estimates Vector: f*, u
0.4	0.75	0.25	0.1500	0.0570	0.3333	0.3330	9.2729	1.0000	1.1560	0.8164
0.4	0.75	0.5	0.3530	0.1340	0.1667	0.1670	8.6374	0.9310	1.0260	0.7246
0.4	0.75	0.75	1.0470	0.3980	0.1111	0.1110	8.4237	0.9080	1.0460	0.7387
0.4	0.75	1.0	2.1790	0.8290	0.0833	0.0830	8.3187	0.8970	1.2550	0.8863
0.6	0.75	0.25	0.1160	0.0440	0.2222	0.2220	8.8492	0.9540	1.0650	0.7521
0.6	0.75	0.5	0.3150	0.1200	0.1111	0.1110	8.4237	0.9080	0.9750	0.6886
0.6	0.75	0.75	0.9840	0.3740	0.0741	0.0740	8.2837	0.8930	1.0060	0.7105
0.6	0.75	1.0	1.7020	0.6470	0.0556	0.0560	8.2118	0.8860	1.1220	0.7924
0.8	0.75	0.25	0.2040	0.0780	0.1667	0.1670	8.6374	0.9310	1.0200	0.7203
0.8	0.75	0.5	0.3970	0.1510	0.0833	0.0830	8.3187	0.8970	0.9540	0.6737
0.8	0.75	0.75	1.0340	0.3930	0.0556	0.0560	8.2118	0.8860	0.9980	0.7048
0.8	0.75	1.0	1.9350	0.7360	0.0417	0.0420	8.1584	0.8800	1.1650	0.8227
1.0	0.75	0.25	0.1370	0.0520	0.1333	0.1330	8.5105	0.9180	0.9890	0.6984
1.0	0.75	0.5	0.3580	0.1360	0.0667	0.0670	8.2553	0.8900	0.9370	0.6617
1.0	0.75	0.75	0.9500	0.3610	0.0444	0.0440	8.1702	0.8810	0.9750	0.6886
1.0	0.75	1.0	2.0170	0.7670	0.0333	0.0330	8.1276	0.8760	1.1780	0.8319

**Table 7:** The values of optimization criteria for the variable parameters of machining at fixed depth of cut—*a_p_* = 1.0 mm.

Variable Parameters			Optimization Criteria							
*x*_1_ Cutting Speed: *v_c_*, (m/min)	*x*_2_ Depth of Cut: *a_p_*, (mm)	*x*_3_ Feed: *f_r_*, (mm/rev)	Surface Roughness: *Ra* (µm)	Dimensionless Surface Roughness: *f*_1_ (Ra*), u	Unit Volume Machining Time: *T_m_* (min/cm^3^)	Dimensionless Volume Machining Time: *f*_2_ (T_m_*), u	Unit Cost Price of Processing One Part: *C*, ($)	Dimensionless Cost Price of Processing One Part: *f*_3_ (C*), u	Length of Estimates Vector: *f*, u	Length of Estimates Vector: f*, u
0.4	1.0	0.25	0.1290	0.0490	0.2500	0.2500	9.2729	1.0000	1.1190	0.7903
0.4	1.0	0.5	0.4400	0.1670	0.1250	0.1250	8.6374	0.9310	1.0100	0.7133
0.4	1.0	0.75	1.0200	0.3880	0.0833	0.0830	8.4237	0.9080	1.0290	0.7267
0.4	1.0	1.0	2.6290	1.0000	0.0625	0.0630	8.3187	0.8970	1.3670	0.9654
0.6	1.0	0.25	0.1890	0.0720	0.1667	0.1670	8.8492	0.9540	1.0400	0.7345
0.6	1.0	0.5	0.4130	0.1570	0.0833	0.0830	8.4237	0.9080	0.9650	0.6815
0.6	1.0	0.75	0.9990	0.3800	0.0556	0.0560	8.2837	0.8930	0.9990	0.7055
0.6	1.0	1.0	1.8840	0.7170	0.0417	0.0420	8.2118	0.8860	1.1580	0.8178
0.8	1.0	0.25	0.1500	0.0570	0.1250	0.1250	8.6374	0.9310	0.9980	0.7048
0.8	1.0	0.5	0.3550	0.1350	0.0625	0.0630	8.3187	0.8970	0.9410	0.6645
0.8	1.0	0.75	1.2140	0.4620	0.0417	0.0420	8.2118	0.8860	1.0200	0.7203
0.8	1.0	1.0	2.0140	0.7660	0.0313	0.0310	8.1584	0.8800	1.1800	0.8333
1.0	1.0	0.25	0.2240	0.0850	0.1000	0.1000	8.5084	0.9180	0.9750	0.6886
1.0	1.0	0.5	0.3250	0.1240	0.0500	0.0500	8.2542	0.8900	0.9260	0.6540
1.0	1.0	0.75	1.0000	0.3800	0.0333	0.0330	8.1695	0.8810	0.9770	0.6900
1.0	1.0	1.0	1.8930	0.7200	0.0250	0.0250	8.1271	0.8760	1.1450	0.8086
